# Scrub Typhus in Children: A Prospective Observational Study in a Tertiary Care Hospital in Eastern India

**DOI:** 10.7759/cureus.41976

**Published:** 2023-07-16

**Authors:** Jadab Kumar Jana, Anusree Krishna Mandal, Soumya Gayen, Dipti Mahata, Md Suhail Alam Mallick

**Affiliations:** 1 Pediatrics, Bankura Sammilani Medical College and Hospital, Bankura, IND

**Keywords:** tropical disease, scrub typhus, complication, co-infection, : children

## Abstract

Background

Scrub typhus is a reemerging, acute, undifferentiating febrile illness and one of the most neglected tropical diseases, calling for an in-depth investigation into its clinical diversity, complications, and mortality, which drives us to carry out this research work.

Methods

Over a year, prospective observational research was carried out after gaining parental consent and institutional ethical clearance, 206 children of either gender aged between one month and 12 years who had been hospitalized with a fever for at least five days and subsequently tested positive for *Orientia*​​​​* tsutsugamushi* were included in the study. Basic demographic information, clinical characteristics, laboratory findings, complications, related coinfections, and results were gathered and analyzed. A *P*-value of 0.05 was set as the statistical benchmark.

Results

The current study found that boys outnumbered girls. The ratio of boys to girls was 1.22:1, and the average age was 5.18 years. All had a fever (100%), and the other most frequently occurring clinical signs and symptoms were abdominal pain (16.99%), vomiting (22.33%), hepatosplenomegaly (49.51%), facial puffiness (39.32%), edema (27.18%), lymphadenopathy (19.90%), eschar (19.90%), macular-erythematous rash (17.96%), cough (21.84%), conjunctival congestion (25.24%), and headache (13.59%). Anemia (81.55%), leucocytosis (20.39%), leucopenia (6.8%), thrombocytopenia (49.51%), thrombocytosis (2.43%), and elevated serum levels of alanine aminotransferase (ALT, 57.28%) and aspartate aminotransferase (AST, 63.59%) were characteristic laboratory results. The coinfections were dengue, enteric fever, urinary tract infections, and malaria. Children who also had dengue were more likely to develop thrombocytopenia, which was statistically significant (*P*-value = 0.008). With doxycycline medication, early defervescence of fever occurred earlier than with azithromycin, and it was statistically significant (*P*-value = 0.000). The complications were hepatitis (63.59%), lower respiratory tract infections (LRTIs, 22.82%),* *scrub typhus meningoencephalitis (STME, 3.88%), acute kidney injury (AKI, 2.91%), myocarditis (1.46%), and acute disseminated encephalomyelitis (ADEM, 0.49%). Except for one who had ADEM, everyone was sent back home after receiving the best care possible. The average duration of hospital stay was 6.89 days.

Conclusions

Even in the absence of eschar, scrub typhus should be suspected in any febrile child who experiences clinical signs of meningoencephalitis syndrome, capillary leakage, skin rash, conjunctival congestion, LRTI, AKI, lymphadenopathy, hepatosplenomegaly, thrombocytopenia, and liver dysfunction in the post-monsoon season. Strong clinical suspicion and prompt anti-scrub drug administration go a long way in preventing or decreasing the morbidity and mortality of the same.

## Introduction

Scrub typhus is endemic in India due to its location in the Tsutsugamushi Triangle. The main contributing factors to its endemicity are increased farming area, livestock, lower education level, an outside cooking place, and lack of proper indoor sanitary facilities. The triangle is bounded by Japan to the north, Northern Australia to the south, and the Arabian Peninsula to the west [1]. Similar infections have been reported from Africa, the Middle East, and South America [2], indicating that it is not limited to this triangle. It is a zoonotic disease, with humans being accidental hosts. The most prevalent rickettsial infection, scrub typhus, is becoming more common in India [3]. Fever, headache, nausea, vomiting, stomach pain, shortness of breath, hepatosplenomegaly, generalized edema, maculopapular rash, and lymphadenopathy are the most typical clinical symptoms in children [4]. Only 11% to 43% of cases with scrub typhus have an eschar, which is its pathognomonic indication [5]. Due to the nonspecific clinical symptoms and seasonal profile shared with leptospirosis and dengue, early diagnosis is frequently challenging [6]. The activity of the mite increases significantly in the monsoon and post-monsoon months. Due to a variety of nonspecific symptoms, low index of suspicion, and lack of knowledge, failure to diagnose it promptly can delay treatment and result in the spread of the bacteria throughout the body, which can lead to the emergence of several complications, including interstitial pneumonia, myocardial infarction, hepatitis, meningoencephalitis, acute respiratory distress syndrome (ARDS), and multiorgan failure [7]. Median mortality of 6% (0%-70%) has been seen when untreated [8]. Therefore, to reduce morbidity and mortality, a high index of suspicion and early empirical antibiotic therapy is crucial. The western region of the Indian state of West Bengal lacks systematic studies on pediatric scrub typhus involving more than 200 children of either gender. Henceforth, this prospective observational study was conducted on the demography, clinical presentation, laboratory features, complications, coinfections, and response to therapeutic measures in children with scrub typhus in a tertiary care teaching hospital.

## Materials and methods

This study was conducted in the Department of Pediatrics, Bankura Sammilani Medical College and Hospital (BSMCH), Bankura, India. The study design is a hospital-based combination of retrospective and prospective cohort studies. The study period spans from January 1, 2022, to December 31, 2022.

Study population

Inclusion Criteria

The inclusion criteria for this study are as follows:

-One-month to 12-year-old children of either gender admitted to the Department of Pediatrics, in the general ward as well as the pediatric intensive care unit (PICU). The age limit is owing to the local administrative policy for pediatric admissions in the corresponding department.

-Presence of fever lasting for five days or more 

-Positive IgM for tsutsugamushi using the enzyme-linked immunosorbent assay (ELISA, with a cut-off of optical density, or OD, >0.5), indicating a diagnosis of scrub typhus fever

-Informed consent provided by either the parents or caretakers of the children

Exclusion Criteria

Children whose parents or caregivers did not provide consent for their child's participation are excluded from the study.

Sample Size

All the consecutive study subjects fulfilling the inclusion criteria over one year in the corresponding study period accounted for the sample size of the study, that is, 206.

Study Technique

In all study participants, relevant information, including age, gender, presenting symptoms, clinical examination findings, complications, coinfections, treatment outcome, and length of hospital stay, was collected in a predesigned proforma that was prepared after pilot testing involving 20 parents (12 mothers and eight fathers) with their kids who were affected by scrub typhus fever in the previous year of the present study period. Automatically, those parents, including their children, were excluded from the present study. Laboratory tests like the complete blood count (CBC), liver function test (LFT), serum urea, creatinine (Cr), serum electrolytes, prothrombin time (PT), urine culture/sensitivity with its bacteriological profile, Widal test, malarial parasite dual antigen (MPDA) kit test, ELISA for dengue IgM, serology for viral hepatitis, and chest X-ray were done. Other imaging studies like ultrasonography (USG) and magnetic resonance imaging (MRI) were advised when indicated. Lumbar puncture and cerebrospinal fluid (CSF) studies were done in some patients when clinically indicated, such as those with meningoencephalitis. All children included in the study population received standard care as per unit protocol, and their treatment outcome was recorded.

Data Analysis

The collected data were put into a Microsoft Excel Sheet and analyzed with the help of Epi-Info (version 3.5.1, Centers for Disease Control and Prevention, Atlanta, GA, USA). Continuous data were expressed as mean and standard deviation, whereas categorical data were expressed as ratios and percentages. Comparison of two or more categorical data was analyzed by either Student's t-tests, chi-square tests, or ANOVA, respectively. A *P*-value <0.05 was set as statistically significant.

Ethical Approval

Institutional ethical approval (Vide memo no. BSM/Aca: 3622, December 9, 2021) was taken before the commencement of the study.

## Results

Demographical characteristics

A total of 206 participants of either gender took part in this research study from January 1, 2022, to December 31, 2022. All participants came from the countryside. Male children aged one month to 12 years made up 54.85% (*n* = 113) of the population, while female children of the same age made up 45.15% (*n* = 93). The male-to-female ratio was 1.22:1. The average age was 5.18 years, with a minimum of one month and a maximum of 11.5 years. The age group of two to five years was more affected (43.2%) than the other two groups, that is, one month to one year (15.0%) and six to 12 years (41.8%). Though male children were infected more than female ones, this is not statistically significant (*P* = 0.093). Demographic characteristics are shown in Table 1.

**Table 1:** Demographic characteristics of the study participants. The *P*-value for gender comparison using the paired sample t-test is 0.093. *Mean ± SD = 5.18 ± 3.17 years; maximum, 11.5; minimum, 0.08. SD, standard deviation

Age*	Gender, *n* (%)		Total, *n* (%)
	Male	Female	
1 month to 1 year	18 (8.7)	13( 6.3)	31(15.0)
2-5 years	50 (24.27)	39 (18.93)	89 (43.2)
6-12 years	45 (21.85)	41(19.90)	86 (41.8)
Total, *n* (%)	113 (54.85)	93 (45.15)	206 (100)

**Figure 1:**
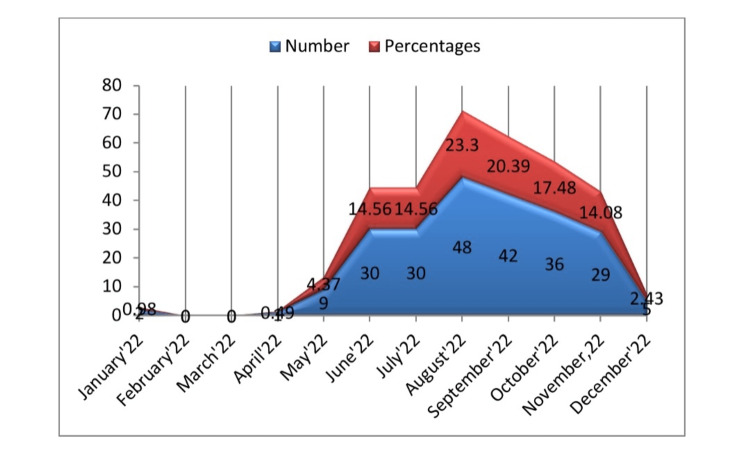
Distinct seasonal variation scrub typhus fever in children.

The current study found that children admitted to the pediatric department had a clear seasonal variation of scrub typhus fever, with a sharp rise from April to July, peaking in August, and then a sharp fall over the following four months, from September to December, giving the characteristic shape of a hill in the plains (Figure 1).

Clinical features

Fever was the most common presenting symptom in all cases. Other nonspecific signs and symptoms were features of capillary leakage: facial puffiness and edema, lymphadenopathy, headache, loss of appetite, and myalgia. The clinical findings that suggested nervous system injury included convulsions, meningeal signs, altered sensorium, and focal neuro deficiency. Other symptoms pointing to a lower respiratory tract infection (LRTI) were cough, crackles, and pleural effusions. In the present study, signs and symptoms of the gastrointestinal system were more common. Other symptoms were hematuria, dysuria, conjunctival congestion, photophobia, maculopapular rash, and wheal. Eschar, the pathognomonic sign of scrub typhus, was present. All the clinical features of scrub typhus fever in children are systematically displayed in Table 2.

**Table 2:** Clinical features of scrub typhus in children.

Signs and symptoms	Number, *n*	Percentage (%)
General		
Fever	100	100
High-grade fever	51	24.76
Edema	56	27.18
Facial puffiness	81	39.32
Lymphadenopathy	41	19.90
Loss of appetite	13	6.31
Myalgia	10	4.85
Headache	28	13.59
Irritability	8	3.88
Shortness of breath	9	4.37
Central Nervous System		
Convulsion	8	3.88
Meningeal signs	6	2.91
Altered sensorium	5	2.43
Focal neurodeficit	1	0.49
Respiratory system		
Cough	45	21.84
Crepitations/crackles	11	5.33
Pleural effusion	3	1.46
Cardiovascular system		
S3 gallop	3	1.46
Pericardial effusion	1	0.49
Gastrointestinal system		
Pain abdomen	35	16.99
Vomiting	46	22.33
Loose stools	13	6.31
Hepatomegaly	55	26.7
Splenomegaly	31	15.04
Hepatosplenomegaly	102	49.51
Ascites	6	2.91
Renal system		
Hematuria	11	5.34
Dysuria	4	1.94
Eyes		
Conjunctival congestion	52	25.24
Photophobia	9	4.37
Skin		
Eschar	41	19.90
Maculoerythematous rash	37	17.96
Ecchymosis	14	6.78
Petechiae	11	5.34
Urticaria/wheal	8	3.88

Laboratory profiles

Table 3 displays the laboratory profiles of the subjects in the present study.

**Table 3:** Laboratory profiles of scrub typhus fever in children. ^*^The mean platelet count in children with scrub typhus and dengue fever was 0.55 × 10^5 ^mm^-3^ and *P*-value = 0.008. ALT, alanine aminotransferase; AST, aspartate aminotransferase

Variables	*n* (%), *N* = 206	Mean ± SD	Number of abnormal value, *n* (%)
Hemoglobin (g/dL)		9.32 ± 1.35	168 (81.55)
≥11	38 (18.45)		
7-˂11	162 (78.64)		
˂7	6 (2.91)		
RBC (×10^6 ^mm^-3^)		4.04 ± 0.44	00 (0.00)
3-5.4	206 (100)		
WBC (×10^3 ^mm^-3^)	11.20 ± 6.13	56 (27.18)	
˃11	42 (20.39)		
4-11	150 (72.82)		
˂4	14 (6.8)		
Platelets^*^ (×10^5 ^mm^-3^)		1.79 ± 1.07	107 (51.94)
˃4.5	5 (2.43)		
1.5-4.5	99 (48.05)		
˂1.5	102 (49.51		
ESR (mm in the first hours)		31.66 ± 14.79	157 (76.21)
≤20	49 (23.79)		
˃20	157 (76.21)		
Liver function test			
ALT (≤40 U/L)	118 (57.28)	7.57 ± 50.39	118 (57.28)
AST (≤40 U/L)	131 (63.59)	70.45 ± 67.93	131 (63.59)
Alkaline phosphatase (145-560 U/L)	206 (100)	183.03 ± 84.67	00 (00.00)
Total protein (g/dL)		6.21 ± 1.03	9 (4.37)
4.2-8	197 (95.63)		
˂4.2	9 (4.37)		
Albumin (g/dL)		3.20 ± 0.53	46 (22.33)
2.8-5.5	160 (76.67)		
˂2.8	46 (22.33)		
Total bilirubin (mg/dL)		0.64 ± 0.55	8 (3.88)
0.2-1	198 (96.12)		
˃1	10 (4.85)		
Renal function test			
Urea (mg/dL)		24.49 ± 10.55	10 (4.85)
˂40	196 (95.15)		
˃40	10 (4.85)		
Creatinine (mg/dL)		0.74 ± 0.25	6 (2.91)
˂1	200 (97.09)		
˃1	6 (2.91)		
Serum electrolytes			
Sodium (mEq/L)		135.57 ± 4.44	75 (36.41)
135-145	131 (63.59)		
˂135	75 (36.40)		
Potassium (mEq/L)		3.82 ± 0.40	31 (15.04)
3.5-5.5	175 (84.95)		
˂3.5	31 (15.05)		

CSF had been analyzed in 8 (3.88%) children (M = 6, F = 2) who were admitted with features of acute encephalitis syndrome, and an abnormal cell count and elevated protein level were detected in 100% (*n* = 8) and 87.5% (*n* = 7) of cases, respectively, and these features suggest aseptic meningitis. The CSF study did not show hypoglycorrhachia, which is frequent in pyogenic and tubercular meningitis. The CSF study is analyzed in Table 4.

**Table 4:** Findings of CSF analysis in children with scrub typhus. CSF, cerebrospinal fluid; SD, standard deviation

Variables	Mean ± SD	Minimum	Maximum	Number abnormal value, *n* (%)
Cell count (0-5 mm^-3^)	42.75 ± 25.25	15	80	8 (100)
Neutrophil	3.38 ± 1.92	0	5	0
Lymphocytes	38.88 ± 52.52	10	70	8 (100)
Biochemical				
Sugar (40-60 mg/dL)	52.5 ± 4.78	46	61	0
Protein (20-40 mg/dL)	68.75 ± 15.18	62	90	7 (87.5)

A chest X-ray was recommended for patients (n = 67) presenting with cough and cold symptoms, and examination revealed crackles. Among these patients, 33 (49.25%) had chest X-rays showing nonspecific infiltration in both lung fields, and 3 (4.48%) showed mild pleural effusion. These findings were later confirmed by thoracic USG. The MRI of the brain was done in all cases of scrub typhus meningoencephalitis (STME, *n* = 8), but only one showed the features of acute disseminated encephalomyelitis (ADEM).

Coinfection

**Figure 2:**
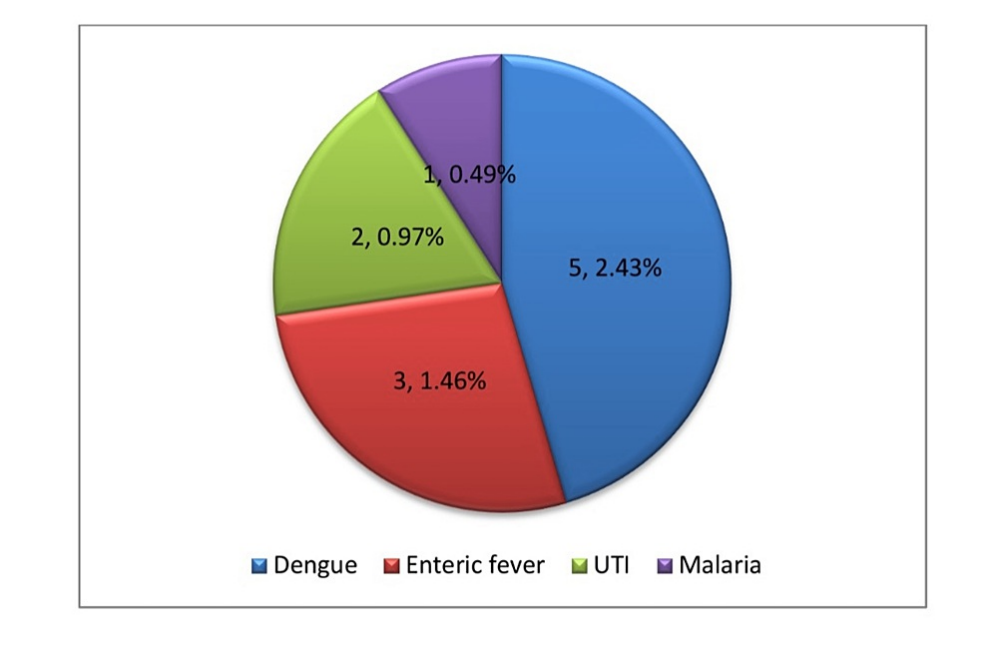
Different types of coinfection with scrub typhus fever in children.

This study showed that 11 (5.34%) participants were coinfected with dengue, enteric fever, urinary tract infection (UTI), and malaria (*Plasmodium falciparum*) in 5 (2.43%), 3 (1.46%), 2 (0.97%), and 1 (0.49%) of study subjects, respectively, as depicted in Figure 2.

Treatment outcome

Except for a few individuals with STME who received a maximum of 10 days of treatment, all subjects had received anti-scrub antibiotics upon suspicion, either with azithromycin (10 mg/kg per day for five days) or doxycycline (4.4 mg/kg in two divided doses for five days). Azithromycin was given to 95 (46.12%) of the 206 participants in the prescribed dose, and the remaining 111 (53.88%) got doxycycline. Our research showed that the doxycycline group experienced early fever defervescence that was statistically significant (*P*-value = 0.000). The variation in clinical response is shown in Table 5.

**Table 5:** Different clinical responses between azithromycin and doxycycline. The *P*-value for comparison of anti-scrub medication using the Student t-test is 0.000.

Name of the anti-scrub medication	Number of participants, *n* (%)	Mean ± SD (hours)
Azithromycin	95 (46.12)	37.43 ± 13.08
Doxycycline	111 (53.88)	27.93 ± 11.69

**Figure 3:**
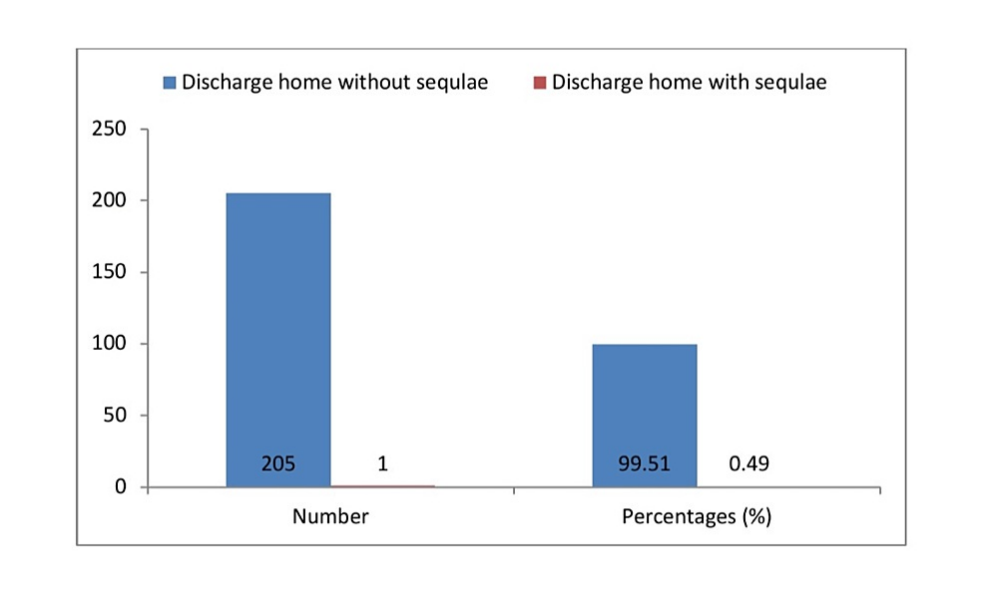
Treatment outcome of scrub typhus in children.

Except for one, all participants (205, 99.51%) were sent home after successful management. The subject who had ADEM was discharged with motor impairment and given the go-ahead for consultations in physical medicine, rehabilitation, and neurological care in the hopes of regaining full functional capacity. Figure 3 shows the treatment outcome.

Complications of scrub typhus

**Figure 4:**
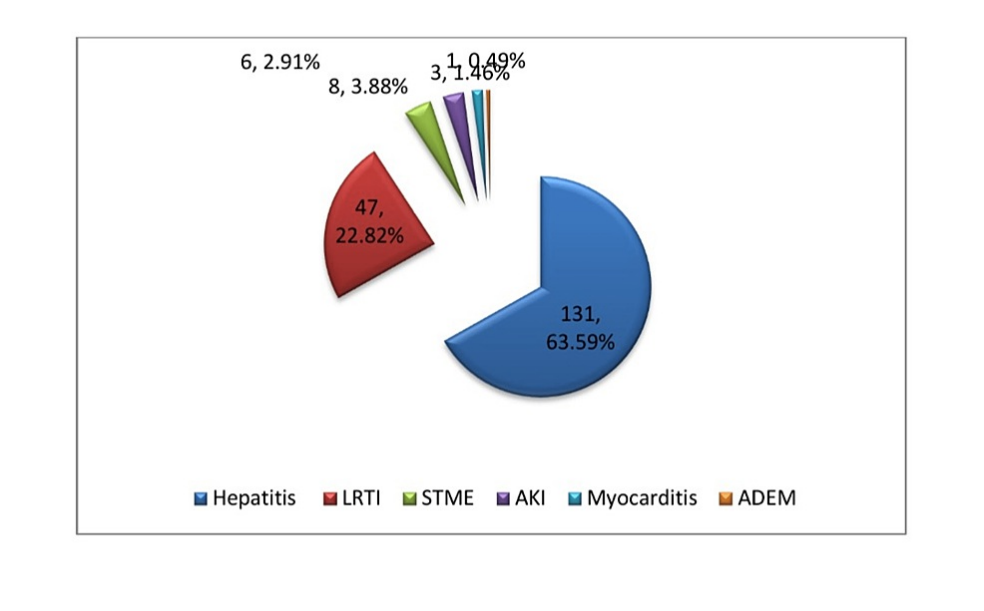
Complications in children with scrub typhus fever. AKI, acute kidney injury; LRTI, lower respiratory tract infection; STME, scrub typhus meningoencephalitis; ADEM, acute disseminated encephalomyelitis

The two most prevalent complications were hepatitis and LRTIs, which were both present in 131 (63.59%) and 47 (22.82%) of the research participants, respectively. Other complications were STME (8, 3.88%), acute kidney injury (AKI; 6, 2.91%), myocarditis (3, 1.46%), and ADEM (1, 0.49%) . The various complications are depicted in Figure 4.

**Figure 5:**
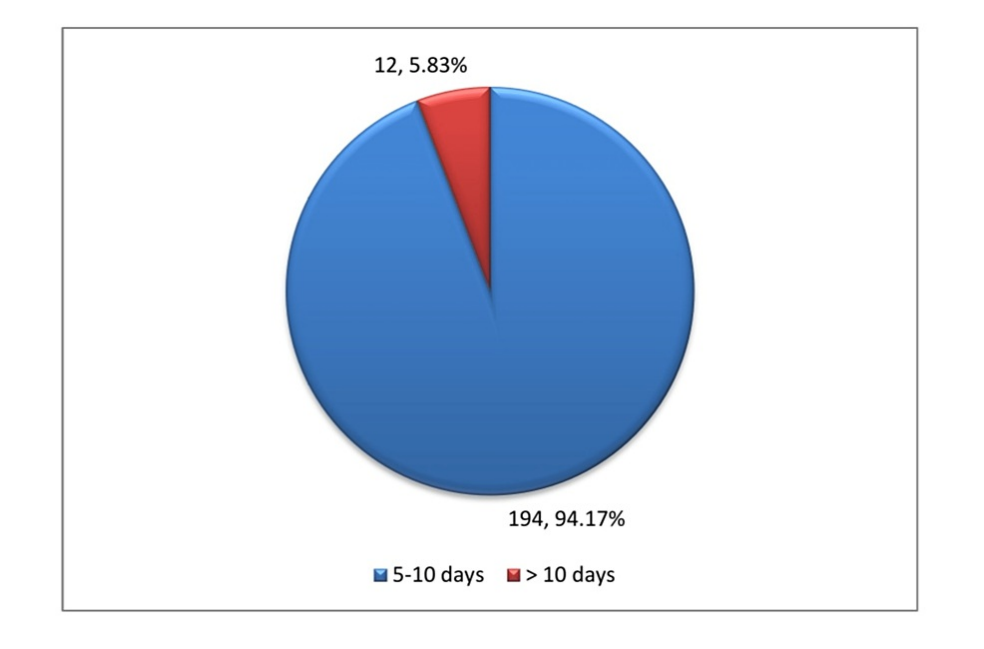
Length of hospital stay.

Only 12 (5.83%) of the 206 patients required more than 10 days of hospitalization before discharge; 194 (94.17%) were sent home between five and 10 days (Figure 5). The average hospital stay was 6.89 days, with the greatest and minimum lengths being 14 and five days, respectively.

## Discussion

Scrub typhus is among the most commonly overlooked and emerging acute undifferentiated febrile illnesses. In their study, Mittal et al. claimed that scrub typhus is widespread throughout India [9]. The western region of West Bengal, an Indian state, has experienced a high incidence of scrub typhus over the previous five years. Many children have been admitted to our hospital with fever for five days or longer and nonspecific signs and symptoms because this referral facility addresses patients from the entire Bankura district, parts of Purulia, West Midnapore, Purba and Paschim Bardhaman, and the neighboring Indian state of Jharkhand. The majority of these kids have developed positive IgM tests for *O. tsutsugamushi*. We observed that the incidence of scrub typhus was higher from June to November, with August seeing the highest number of cases. The largest number of cases was observed between September and February, which are believed to be cooler months, according to Varghese et al. [10]. However, Weitzel et al. reported that all cases of scrub typhus occurred between January and February in research done in South America, suggesting that summer may be the time of year when vector activity is the highest [11]. Scrub typhus in children has a wide range of clinical characteristics and a typical seasonal variation similar to other tropical diseases such as enteric fever, malaria, leptospirosis, and dengue. To save the child's life going forward, strong clinical suspicion, consideration of seasonal change, and prompt delivery of anti-scrub measures are crucial. There were 206 participants in this study, of either gender, ranging in age from one month to 12 years. Out of 206 participants, 113 (54.85%) were males and 93 (45.15%) were females. It was 1.22 boys for every girl. Participants were 5.15 years old on average. The younger boy was older than all the others, being 11.5 years old, while the youngest girl was just 0.125 years old (45 days). Two- to five-year-olds made up 43.2% (*n* = 89) of the age group that was most frequently affected, which could be explained by their greater propensity for outdoor play and general curiosity about the universe. The demographic characteristics of the present study participants were identical to those in a study by Agrawal et al. [12]. In contrast to the current study, a study by Kumar et al. [13] found that the mean age of presentation was 10.63 years. However, their study covered children up to the age of 18, whereas the present study adhered to state administrative regulations and only included children between the ages of one month and 12 years. In the present study, despite keeping infants indoors, 15% of the total study participants were affected. This indicates that the habitat of the chiggers has progressively encroached closer to the residents' houses, highlighting the need for effective measures to address the situation. According to prior research from India [14], fever (five days or more) was the primary complaint in every case. High-grade fever was found in 51 (24.76%) out of 206. Other nonspecific overlapping clinical features with dengue and enteric fever were edema (56, 27.18%), facial puffiness (81, 39.32%), lymphadenopathy (41, 19.9%), loss of appetite (13, 6.31%), myalgia (10, 4.85%), headache (28, 13.59%), shortness of breath (9, 4.37%), and irritability (8, 3.88%). A study from Kolkata, India, conducted by Dasgupta et al. [15] revealed that edema was present in 26.3% of patients, which is consistent with the current findings. The current study found lymphadenopathy in 41 (19.9%) participants. It has been documented in 10% to 62% of cases by various authors [13,16,17]. In the current investigation, headache was present in 28 (13.59%) cases, which contrasts with the finding of 28.7% in the study by Kumar et al. [13]. This discrepancy results from the two studies' different mean ages. The mean age of the population in this study was 5.15 years compared to the mean age of 10.63 years reported in a study by Kumar et al.. Given that the elder child can verbally communicate their complaint, the smaller youngster cannot.

The most frequent gastrointestinal symptoms noted in this study were abdominal pain (35, 16.99%), vomiting (46, 22.33%), loose motion (13, 6.31%), hepatomegaly (55, 26.7%), splenomegaly (31, 15.04%), hepatosplenomegaly (102, 49.51%), and ascites (6, 2.91%). These gastrointestinal signs and symptoms, except ascites and loose motion, were also observed by Agrawal et al. from Odisha, India, and Pathak et al. from Nepal, but with a different frequency [12,18]. A study from Odisha, India, conducted by Mallick et al. revealed ascites and demonstrated that it was prevalent in 15% of their study population, which is consistent with the current study [19], although Dasgupta et al. stated that the same was present in 24.4% of their study participants [15]. Researchers Kumar et al. from Pondicherry and Krishnan et al. from Kerala, India, conducted two studies on scrub typhus in children, finding loose motion in 11% and 10.18% of their study participants, respectively [16,20]. The present study, which found 24 (11.65%) participants, just suppresses these findings.

As indicators of neurological involvement, convulsion, meningeal symptoms, altered sensorium, and focal neurodeficiency were found in eight (3.88%), six (2.91%, five (2.43%), and one (0.49%) participants, respectively. In their study from Odisha, India, Agrawal et al. revealed a greater frequency of seizures (11.5%) and altered sensorium (9.6%) [8]. It can be the result of a delayed admission to the hospital, a delayed diagnosis, or both. In the present study, respiratory findings, including cough, crackles, and pleural effusions, were present in 45 (21.84%), 11 (5.33%), and three (1.46%) cases, respectively. Other authors also observed the respiratory findings of cough, crackles, and pleural effusions [16,21].

In the present study, hematuria and dysuria were observed in 11 (5.34%) and 4 (1.94%) participants, respectively. Hematuria may be brought on by thrombocytopenia, coagulopathy, or UTI that is already evident. According to the present research, two children had UTIs, one of which was caused by *Staphylococcus aureus *and the other by *Escherichia coli*. To the best of our knowledge, there has not been a study that describes UTI as a coinfection in children.

Conjunctival congestion and photophobia were observed in 52 (25.24%) and 9 (4.37%) children in this present investigation, respectively. This study's observation on conjunctival congestion in the eye (52, 25.24%) is consistent with a study from Kolkata by Basu et al. that found 24.6% [22]. The prevalence of conjunctival congestion varied greatly, from 8.3% to 46% [17,23-24].

Maculopapular rash, wheals or hives, and *eschar*, which is the pathognomonic sign of the disease, were the three skin manifestations of scrub typhus and were present in 41 (19.90%), 37 (17.96%), and eight (3.88%) participants, respectively. Eschar helps with the clinical diagnosis of scrub typhus with greater specificity (98.9%), but its usefulness is limited due to a large difference in distribution (7% to 97%) among patients [25,26]. Clinical examinations might be challenging because of dark complexions, unusual eschar presentations, and hidden body parts such as the groin, axillary skin folds, and behind the ear lobule. From this point forward, if the researchers do not rigorously search an individual for it, they will probably overlook it. Wheals or hives, the distinctive skin manifestation, were seen in 8 (3.88%) participants in the current study, leading the doctor to mistake them for acute urticaria from a variety of causes, including malaria [27]. Further research is required to fully understand whether it exists in scrub typhus.

The hematological abnormalities identified in the current study were anemia, leukocytosis, leukopenia, thrombocytosis, thrombocytopenia, and elevated ESR, which were present in 168 (81.55%), 42 (20.39%), 14 (6.8%), 5 (2.43%), 102 (49.51%), and 157 (76.21%) cases, respectively. The percentage of anemia associated with scrub typhus reported in several studies conducted by various authors from different parts of India was between 29% and 80% [15,28]. Palanivel et al. [29] from Chennai, India, revealed that 83% of the people they studied had anemia, which is consistent with the findings of the present study. Higher percentages of anemia in this research could be the result of underlying nutritional anemia or the disease itself, necessitating more investigation. In the majority of patients (150, 72.82%), the total leukocyte count was normal. However, the current study's leukocytosis rate of 20.39% was very similar to that of a study by Kispotta et al. that found 17.1% [28]. Other researchers also reported leukocytosis [17,29,30]. Leukopenia is another abnormality of the white blood cells reported in scrub typhus and was detected in 14 (6.8%) cases in the current investigation. Children with scrub typhus experience leukopenia in between 0.92% and 27% of cases, according to studies by different authors [4,20,31]. A total of 102 (49.51%) participants in the present study had thrombocytopenia. According to many studies [16,20,23,24], it varies from 26% to 100%. Immune thrombocytopenia may be the cause of thrombocytopenia in scrub typhus [32]. Reactive thrombocytosis, which is another kind of thrombocytosis, was seen in five (2.43%) study samples. An increase in serum thrombopoietin due to thrombocytopenia stimulates the megakaryocytes in the bone marrow and raises the number of platelets in the blood [33].

Scrub typhus fever is associated with some biochemical alterations, including elevated levels of ALT, AST, and bilirubin as indicators of liver damage; hypoproteinemia and hypoalbuminemia as indicators of either capillary leakage or diminished liver function; uremia and creatinemia as markers of AKI; and hyponatremia and hypokalemia as indicators of either nutritional deficiency or diminished kidney function. In the current research, ALT and AST levels rose in 118 (57.28%) and 131 (63.59%) participants, respectively. In 8 (3.88%) patients, serum bilirubin levels rose. The levels of urea (10, 4.8%) and creatinine (6, 2.91%) also increased in 10 (4.8%) and 6 (2.9%) cases, respectively. What we saw in the present study was also seen in several studies conducted by authors around the world, albeit at varied frequencies, due to abnormal liver and renal functioning [12,13,19,20,21,28].

After ruling out all potential contraindications, 8 (3.8%; M = 6, F = 2) children presenting with acute encephalitis syndrome underwent a CSF examination in the present study. Abnormal cell counts and increased protein levels were found in 100% and 87.5% of all meningoencephalitis cases, respectively, and these characteristics suggest aseptic meningitis. Hypoglycorrhachia, which is usually identified in pyogenic and tubercular meningitis, was not detected in the CSF analysis of the current research. The mean CSF cell counts (42.75 mm^-3^), sugar (52.5 mg/dL), and protein (68.75 mg/dL) in the present study are comparable to those in the studies conducted by Roy et al. and Meena et al. [34,35].

Hepatitis was identified in 131 (63.59%) children in the present study, and icteric hepatitis made up 3.8% (n = 8). In their study, Palanivel et al. [29] of Chennai, India, reported 64% hepatitis, which was consistent with the current study. Hepatitis was also found in scrub typhus reported by several writers; however, the incidences ranged from 31% to 77% [16,18,30,36]. There were 16.19% (n = 33), 3.88% (n = 8), 2.91% (n = 6), 1.46% (n = 3), and 0.49% (n = 1) other complications of LRTI, STME, AKI, myocarditis, and ADEM, respectively. In their study population from Odisha, Agrawal et al. [12] reported 20.6% LRTI, which was slightly higher than the results of the current study. In contrast to the current study, Khemka et al. [37] observed 67.7% of respiratory distress due to a different study location. Khemka et al. carried out their study in a PICU equipped with top-of-the-line facilities for seriously ill children. In the current study, 3.88% (n = 8) of the study population had STME. According to Silpapojakul et al. [38], 3% of the study population developed STME, which was compatible with the results of the present study. Various publications have found an incidence of STME that ranges from 3% to 34.4% [22,38]. The highest (34.4%) was reported by Basu et al. from Kolkata, a metropolitan city-based tertiary healthcare facility in the Indian state of West Bengal, and many children were referred there from rural-based tertiary care facilities like the present study area for super-specialized services. In comparison to earlier studies, which ranged from 4.3% to 65.8% [12, 13-18], the present study's finding of AKI was 2.91% (n = 6), a noticeably lower figure. The highest frequency was observed (65.8%) [18] by Pathak et al. from Nepal. It could be brought on by late arrival at a medical facility, delayed diagnosis, delayed execution of anti-scrub measures, a person's genetic predisposition, or genetic variation of *O. tsutsugamushi*. Another scary complication is myocarditis, which can raise fatality rates if it is not diagnosed and treated promptly. Myocarditis was observed in the current study with a frequency of 1.46% (n = 3), which was also reported by other authors with a range of a frequency between 4.8% and 34% [12,16,17]. In their study, Roy et al. [34] identified ADEM-like characteristics in 0.74% of cases, which was comparable to the current study's description of one ADEM with scrub typhus. There is an increasing need for research into the precise pathophysiology of ADEM in scrub typhus.

The coinfection mentioned here was another key component of the present investigation. In the present study, scrub typhus co-occurred with dengue, enteric fever, UTIs, and malaria in five (2.43%), three (1.46%), two (0.97%), and one (0.49%) cases, respectively. Basu et al. reported that 2.12% of their research population had dengue fever as a coinfection [22]. As far as we are aware, no previous investigation has identified enteric fever, UTIs, and malaria as coinfections with scrub typhus in the pediatric population.

Doxycycline is the antibiotic of choice for scrub typhus fever. It should be administered either orally or intravenously in doses of 100 mg twice daily for children over 40 kg and 2.2 mg/kg twice daily for children under 40 kg for three days after the fever has subsided, for a total of seven days. Ten days of therapy may be necessary for severe and challenging occasions. Chloramphenicol, rifampicin, and macrolides (oral clarithromycin or oral or intravenous azithromycin) are other useful medications [39]. In the present study, 111 (53.88%) children received doxycycline, whereas the remaining 95 (46.12%) received azithromycin. In comparison to patients treated with azithromycin (mean = 37.43 hours), patients who received doxycycline experienced early defervescence of fever (mean = 27.93 hours). The difference in outcome was statistically significant (*P*-value = 0.000). Despite the early defervescence of fever with doxycycline, azithromycin had been used in many cases (95, 46.12%) due to its availability in oral suspension, ease of administration in young infants in appropriate doses, and ampoule supply in government-run healthcare facilities. However, there were some drawbacks, such as its unavailability in injectable preparation, which is required for patients with altered sensorium and those with poor oral tolerance. Doxycycline has also demonstrated a comparable clinical response in earlier investigations [21,40].

Similar to the investigations conducted by Digra et al. and Roy et al. [17,34], the present research reported 0% mortality. According to many studies carried out by other authors [12,13], mortality rates ranged from 1% to 12.2%. The likelihood of complications, early detection, appropriate treatment, and ongoing surveillance could all affect the death rate.

In the current study, 194 (94.17%) participants were sent home within five to 10 days. In contrast, only 12 (5.83%) children required more than 10 days in the hospital to make a full recovery, except for one child who had ADEM. The t-test was performed, and the result showed a statistically significant *P*-value = 0.000. The average length of stay in the hospital was 7.64 days. The present study is consistent with the hospital stays reported by Agrawal et al. and Das et al. from Odisha, India, who reported the average duration of hospital stays of 7.2 and 4.6 days, respectively [12,14].

Special case

A five-year-old child was identified as distinctive in the present study and needed a unique description. The child was admitted to the PICU with a five-day high-grade fever, a two-day altered sensorium, and paralysis in both lower limbs that had started just one day before. The child experienced two episodes of generalized tonic-clonic fits, lasting roughly three minutes each within 15 minutes right away after being admitted. The child was stabilized and treated according to unit policy. All required tests, such as an MRI of the brain and a serology against *O. tsutsugamushi*, were encouraged. It took place for the Tsutsugamushi IgM test to be positive, and an MRI of the brain revealed multifocal nodular T2 flair hyperintensity in both cerebral hemispheres, which suggested ADEM. Methylprednisolone (30 mg/kg per day intravenously for five days) and intravenous doxycycline were also given, as well as prednisolone (2 mg/kg per day in divided dosages) for six weeks with tapering. Within 48 hours, the fever declined and the motor weakness progressively became better. With recommendations for routine follow-up in the departments of physical medicine and rehabilitation and neurology, the kid was discharged on day 21 despite having some residual motor weakness in both lower limbs. We learned from this child's presentation that scrub typhus fever can have ADEM-like symptoms; hence, early empirical anti-scrub measures should be encouraged while waiting for the confirming serological test.

Limitations of the present study

It was a study conducted in a hospital. It is, therefore, not devoid of selection bias. How much the C-reactive protein changed and in what proportion it was present in children with scrub typhus fever could not be stated here due to the insufficient data used to measure it.

Strengths of the present study

There were few studies describing the results of systemic examinations over and above 200 children ranging in age from one month to 12 years. The current study involved 206 children of either gender and was a prospective observational study with no casualties over a year. This study also reported an unusual relationship between scrub typhus fever and ADEM. Additionally, we noted reactive thrombocytosis and wheals or hives in 5 (2.43%) and 8 (3.88%) cases, respectively. The coinfections of enteric fever, UTI, and malaria were also described in the present research.

## Conclusions

Scrub typhus fever has a wide range of clinical manifestations, making it difficult for both primary care physicians and pediatricians to distinguish it from other tropical diseases with similar clinical features and seasonal variations. The present study found the disease to be more prevalent in the male gender and the age group of two to five years. Fever was the most common clinical feature along with signs and symptoms of capillary leak, hepatosplenomegaly, and lymphadenopathy. The most common laboratory manifestations were of thrombocytopenia, deranged LFTs, hypoalbuminemia, and hyponatremia. There was no mortality in the present study, though one patient was left with motor impairment as sequelae. Maintaining a high index of clinical suspicion and promptly administering anti-scrub medication are vital. The following other suggestions may be made: augmentation of knowledge through literature review, ensuring the availability of testing (IgM against *O. tsutsugamushi*) in all healthcare facilities, prompt referral following administration of the first dose of anti-scrub medication, prioritizing personal protection, implementing environmental modification strategies, employing vector control measures, increasing research for the development of point-of-care testing with high sensitivity and specificity, and Investing in vaccine research and development.
